# Wideband Spectrum Sensing Using Modulated Wideband Converter and Data Reduction Invariant Algorithms

**DOI:** 10.3390/s23042263

**Published:** 2023-02-17

**Authors:** Gilles Burel, Emanuel Radoi, Roland Gautier, Denis Le Jeune

**Affiliations:** 1 Univ Brest, CNRS, Lab-STICC, CS 93837, 6 avenue Le Gorgeu, CEDEX 3, 29238 Brest, France; 2 ENSTA Bretagne, CNRS, Lab-STICC, 2 rue François Verny, CEDEX 9, 29806 Brest, France

**Keywords:** Xsampling, modulated wideband converter, compressed sensing, data reduction, OMP algorithm, LASSO algorithm, wideband spectrum sensing

## Abstract

Wideband spectrum sensing is a challenging problem in the framework of cognitive radio and spectrum surveillance, mainly because of the high sampling rates required by standard approaches. In this paper, a compressed sensing approach was considered to solve this problem, relying on a sub-Nyquist or Xsampling scheme, known as a modulated wideband converter. First, the data reduction at its output is performed in order to enable a highly effective processing scheme for spectrum reconstruction. The impact of this data transformation on the behavior of the most popular sparse reconstruction algorithms is then analyzed. A new mathematical approach is proposed to demonstrate that greedy reconstruction algorithms, such as Orthogonal Matching Pursuit, are invariant with respect to the proposed data reduction. Relying on the same formalism, a data reduction invariant version of the LASSO (least absolute shrinkage and selection operator) reconstruction algorithm was also introduced. It is finally demonstrated that the proposed algorithm provides good reconstruction results in a wideband spectrum sensing scenario, using both synthetic and measured data.

## 1. Introduction

The research work presented in this paper is mainly related to the wideband spectrum sensing problem, which consists of detecting the occupied or active frequency bands, at a given moment and in a given place, over a very large frequency domain (e.g., larger than 1 GHz). This information is necessary for cognitive radio systems [1] but also for some spectrum monitoring-related civil and military applications [2,3].

In this framework, standard spectral analysis methods resulted in heavy or impractical spectrum sensing architectures because of the very high sampling frequency required and the huge quantity of data to be processed. Since a finite-dimensional signal with a sparse or compressible representation can be recovered exactly from a small set of linear, non-adaptive measurements [4], the compressed sensing approach [5,6,7,8] allows the input sampling constraint to be relieved by taking advantage of the spectrum sparsity [9]. The new constraint is then that at a given time and in a given location, only a small part of the whole monitored frequency band is really occupied. Hence, by taking advantage of this spectrum sparsity, instead of first sampling at a high rate and then compressing the sampled data before processing, the data can be directly sensed at a lower sampling rate in a compressed form.

A recent survey of wideband spectrum sensing approaches with special attention paid to approaches that utilize sub-Nyquist sampling techniques can be found in [10], and ref. [11] provides an overview of recent advances in this domain.

**Figure 1:**
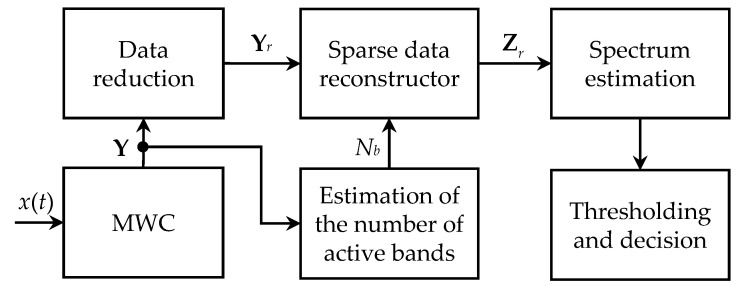
Signal and data processing block diagram for wideband spectrum sensing.

The general wideband spectrum sensing scheme considered in this paper is given in Figure 1. The first stage of the processing chain is represented by the MWC (modulated wideband converter) [12,13], which is able to sample the received signal at a much lower rate than the Nyquist limit ($F_{Nyquist}$), without any information loss, provided that its frequency content is sparse enough. This specific Xsampling method is considered here just because it has been actually used to obtain the experimental results discussed in Section 5, but it is worth noting that some other competing techniques have been also proposed over these last few years.

Thus, ref. [14] describes and discusses an Xsampling architecture named analog-to-information converter (AIC), which aims at acquiring efficiently wideband signals. A blind sub-Nyquist sampling approach, referred to as the quadrature analog-to-information converter (QAIC), is proposed in [15]. It relaxes the analog frontend bandwidth requirements at the cost of some added complexity compared to MWC for an overall improvement in sensitivity and energy consumption. A novel random triggering-based modulated wideband compressive sampling (RT-MWCS) method is also proposed in [16] to facilitate the efficient realization of sub-Nyquist rate compressive sampling systems for sparse wideband signals. Compared to MWC, RT-MWCS has a simple system architecture and can be implemented with one channel at the cost of more sampling time. As a last example, a single channel modulated wideband converter (SCMWC) scheme for the spectrum sensing of band-limited wide-sense stationary (WSS) signals was introduced in [17]. With one antenna or sensor, this scheme can save not only sampling rates but also hardware complexity.

Since the contribution presented in this paper is independent of the type of Xsampling scheme, the MWC architecture was selected for the reason mentioned above. It consists of *M* identical parallel signal processing paths, with a wideband input signal *x*(*t*) on each of them being multiplied with a different $T_{p}$-periodical binary random signal, low-pass filtered, sampled at $F_{s}\ll F_{Nyquist}$, and analog to digital converted. Note that $F_{s}$, the most often equal to $1/T_{p}$, is also twice the cut-off frequency of the low-pass filter.

The MWC output then consists of an *M* × *N* matrix **Y**, where *N* is the number of samples required to ensure a given spectral resolution and *M* ≤ *N*.

The matrix **Y** could be directly used as an input for the sparse data reconstructor, which can be solved with the equation below:

(1) $$Y=W^{H}Z,$$

under the sparsity hypothesis for the expected solution, i.e., the *L* × *N* matrix **Z**. The *L* × *M* matrix **W** involved in Equation (1) is known, its elements being calculated directly from the periodical binary random signals (scramblers) used by the MWC.

In the compressed sensing approach, the price to pay for the reduction in the input sampling rate is the additional processing required by the signal recovery. The dimension of the input matrix **Y** for reconstruction is then of particular importance. Since typical values of *N* may be very large, it is proposed to reduce the data matrix **Y** before sparse data reconstruction. Compared to state-of-the-art published research ([18,19,20]), our approach directly exploits the intrinsic sparsity of the matrix **Y**. Hence, rather than using it as input for the sparse data reconstructor, the following matrix is considered instead:

(2) $$Y_{r}=YV_{Y},$$

where $V_{Y}$ is the *N* × *M* matrix provided by the “economy size” singular value decomposition (SVD) of the matrix **Y**, i.e.:

(3) $$Y=U_{Y}S_{Y}V_{Y}^{H}.$$

In this way, the sparse data reconstructor will work with an *M* × *M* instead of an *M* × *N* input matrix, which considerably reduces the computational burden for large values of *N*. Actually, problem (1) can be rewritten as follows:

(4) $$Y_{r}=W^{H}Z_{r},$$

where $Z_{r}=ZV_{Y}$.

Note that the sparse data reconstructor also requires the estimation of the number of active frequency bands $N_{b}$. This task can be carried out by different algorithms, such as the information-theoretic criteria [21], which makes use of the *M* singular values of **Y** provided by the diagonal of the $S_{Y}$ matrix.

The reduced data matrix $Y_{r}$ and the estimated number of active frequency bands $N_{b}$ are then used in the next stage to find out the sparse problem solution $Z_{r}$ through a greedy algorithm, such as OMP (orthogonal matching pursuit) [22,23], or an optimization-based one, such as LASSO (least absolute shrinkage and selection operator) [24,25]. In [26], the authors showed that LASSO was a suitable choice for compressive spectrum sensing and recovery in wideband 5G cognitive radio networks.

As will be demonstrated in this paper, greedy algorithms are already data reduction invariant and do not require any modification when being used in this framework. However, the standard LASSO algorithm does not have this useful property because of the standard $l_{1}$ norm, which is involved in the optimization process.

In order to overcome this drawback of the standard LASSO algorithm, a new data reduction invariant $l_{1}$ norm was first introduced to replace the standard $l_{1}$ norm in the optimization process. Then, it was demonstrated that the newly defined version of the LASSO algorithm became data reduction invariant. To the best of our knowledge, this is the only data reduction invariant version of the LASSO algorithm proposed so far in the literature.

Finally, once the matrix $Z_{r}$ is provided by the sparse data reconstructor, the input signal spectrum is estimated, and the threshold is determined in order to make a decision about the active frequency bands.

The rest of the paper is organized as follows. In Section 2, it is demonstrated that greedy algorithms for sparse signal reconstruction are already data reduction invariant. The new version of the LASSO algorithm was introduced in Section 3, and its invariance with respect to data reduction was also demonstrated. The performance of the proposed algorithm is finally evaluated using both simulated and measured data in Section 4 and Section 5 respectively, while Section 6 summarizes the research work presented in this paper and provides some conclusions about its results. Some mathematical preliminaries are also provided in Appendix A; the standard OMP algorithm is briefly recalled in Appendix B, while the data reduction invariance of the newly defined $l_{1}$ norm is demonstrated in Appendix C.

The general notations used in this paper are as follows. Matrices and vectors are denoted by symbols in boldface, including uppercase for matrices and lowercase for vectors. ${\left(.\right)}^{T}$ and ${\left(.\right)}^{H}$ represent complex transpose and Hermitian operators, respectively. $I_{M}$ denotes the *M* × *M* identity matrix. ${\left\|\cdot \right\|}_{k}$ and ${\left\|\cdot \right\|}_{F}$ stand for the $l_{k}$ norm and the Frobenius norm, respectively. Some other specific notations are defined in the next sections.

## 2. Data Reduction Invariance of Greedy Algorithms

In this section, it is shown that greedy reconstruction algorithms are invariant with respect to data reduction. Although the invariance property is demonstrated for the OMP algorithm only, this result can be extended by similarity to the other algorithms of this class, such as a compressive sampling matching pursuit (CoSaMP) [27,28] or Iterative Hard Thresholding (IHT) [8,29]. Also note that while an extensive comparison between the proposed algorithm and OMP is carried out in the next section, in terms of the mean square error and detection probability, some results obtained by the CoSaMP and IHT algorithms on measured data are provided as well in Section 4.

Let us consider the two problems corresponding to the original and reduced data matrix, respectively:

(5) $$\begin{array}{l} Y=W^{H}Z,\\ Y_{r}=W^{H}Z_{r}, \end{array}$$

where $Y_{r}=YV_{Y}$ and $Z_{r}=ZV_{Y}$, as already mentioned above.

Let us also define the following notations:

- $J_{k}$: a subset of {1,…,L} with $0\leq card\left\{J_{k}\right\}\leq M$ formed by the indices of non-zero rows of the solution ${\hat{Z}}_{k}$, at the *k*th iteration;
- $W_{\left(k\right)}^{H}$: the $M\times card\left\{J_{k}\right\}$ matrix formed with the columns of $W^{H}$ whose indices belong to $J_{k}$;
- ${\hat{Z}}^{\left(k\right)}$: the $card\left\{J_{k}\right\}\times N$ optimized matrix;
- $\Pi_{Y}=V_{Y}V_{Y}^{H}$.

It can be readily noticed that the matrix $\Pi_{Y}$ is a projector, since $\Pi_{Y}^{2}=\Pi_{Y}^{}\Pi_{Y}^{}=V_{Y}\underset{I_{M}}{\underset{︸}{V_{Y}^{H}V_{Y}}}V_{Y}^{H}=V_{Y}V_{Y}^{H}=\Pi_{Y}^{}$.

Let us finally denote by Fix{Y} the set of all matrices **A** invariant with respect to $\Pi_{Y}$ (fixed points of the projector), so that:

(6) $$A\in Fix\left\{Y\right\}\Leftrightarrow A\Pi_{Y}=A$$

For the first problem in Equation (5), the residual can be written as follows:

(7) $$R^{\left(k\right)}=Y-W_{\left(k\right)}^{H}{\hat{Z}}^{\left(k\right)},$$

where:

(8) $${\hat{Z}}^{\left(k\right)}={\left(W_{\left(k\right)}^{}W_{\left(k\right)}^{H}\right)}^{-1}W_{\left(k\right)}^{}Y=\underset{{\hat{Z}}_{r}^{\left(k\right)}}{\underset{︸}{{\left(W_{\left(k\right)}^{}W_{\left(k\right)}^{H}\right)}^{-1}W_{\left(k\right)}^{}U_{Y}S_{Y}}}V_{Y}^{H}\overset{Lemma\;2}{\Rightarrow }{\hat{Z}}^{\left(k\right)}\in Fix\left\{Y\right\}.$$

Since Y∈Fix{Y}, according to Lemma A1 and Lemma A3 (see Appendix A), the Equations (7) and (8) result in:

(9) $$R^{\left(k\right)}\in Fix\left\{Y\right\}.$$

For the second problem in Equation (5), the residual can be written as follows:

(10) $$R_{r}^{\left(k\right)}=Y_{r}-W_{\left(k\right)}^{H}{\hat{Z}}_{r}^{\left(k\right)}=YV_{Y}-W_{\left(k\right)}^{H}{\hat{Z}}_{r}^{\left(k\right)},$$

where:

$${\hat{Z}}_{r}^{\left(k\right)}={\left(W_{\left(k\right)}^{}W_{\left(k\right)}^{H}\right)}^{-1}W_{\left(k\right)}^{}Y_{r}=\underset{{\hat{Z}}^{\left(k\right)}}{\underset{︸}{{\left(W_{\left(k\right)}^{}W_{\left(k\right)}^{H}\right)}^{-1}W_{\left(k\right)}^{}Y}}V_{Y}^{}\Rightarrow R_{r}^{\left(k\right)}=YV_{Y}-W_{\left(k\right)}^{H}{\hat{Z}}_{}^{\left(k\right)}V_{Y}=\underset{R^{\left(k\right)}}{\underset{︸}{\left(Y-W_{\left(k\right)}^{H}{\hat{Z}}_{}^{\left(k\right)}\right)}}V_{Y}$$

(11) $$\Rightarrow \left\{ \begin{array}{l} {\hat{Z}}_{r}^{\left(k\right)}={\hat{Z}}^{\left(k\right)}V_{Y}\\ R_{r}^{\left(k\right)}=R^{\left(k\right)}V_{Y} \end{array}\right..$$

Since it has already been shown that ${\hat{Z}}^{\left(k\right)},R^{\left(k\right)}\in Fix\left\{Y\right\}$, multiplying Equation (11) at the right side by $V_{Y}^{H}$ results in:

(12) $$\left\{ \begin{array}{l} {\hat{Z}}_{r}^{\left(k\right)}V_{Y}^{H}={\hat{Z}}^{\left(k\right)}\Pi_{Y}\\ R_{r}^{\left(k\right)}V_{Y}^{H}=R^{\left(k\right)}\Pi_{Y} \end{array}\right.\Rightarrow \left\{ \begin{array}{l} {\hat{Z}}_{r}^{\left(k\right)}V_{Y}^{H}={\hat{Z}}^{\left(k\right)}\\ R_{r}^{\left(k\right)}V_{Y}^{H}=R^{\left(k\right)} \end{array}\right.,$$

and finally:

(13) $$\left\|R\right\|=\left\|R_{r}\right\|.$$

Hence, reducing the data matrix does not modify the residual norm. Consequently, taking into account the bijective relationship (11) between ${\hat{Z}}^{\left(k\right)}$ and ${\hat{Z}}_{r}^{\left(k\right)}$, since the OMP algorithm aims at minimizing the residual norm, it can operate as well on the reduced data matrix without changing the final result.

Furthermore, the key elements involved in the OMP algorithm are the scalar products between the $W^{H}$ matrix columns and the residual. More precisely, the relevant information is contained in the diagonal of the matrix below:

$$(WR){\left(WR\right)}^{H}=\left(WR_{r}V_{Y}^{H}\right)\left(WR_{r}V_{Y}^{H}\right)=WR_{r}\underset{I_{M}}{\underset{︸}{V_{Y}^{H}V_{Y}^{}}}R_{r}^{H}W^{H}$$

(14) $$\Rightarrow (WR){\left(WR\right)}^{H}=\left(WR_{r}\right){\left(VR_{r}\right)}^{H}.$$

Hence, this matrix does not change when using a reduced data matrix instead of the original one. Consequently, there exists an isomorphism between the intermediate calculations required by the OMP algorithm running on the two data matrices since all the intermediate variables are linked by bijective relationships, and all the elements involved in the decision-making steps (i.e., residual norm and scalar products) are invariant.

## 3. Data Reduction Invariant Version of LASSO Algorithm

A new version of the LASSO algorithm, invariant to data reduction, is introduced in this section. A key point to keep in mind is that it operates on the reduced data matrix, as explained in the previous section, and therefore, it optimizes $Z_{r}$ instead of Z, which results in a significant complexity reduction. Since $Z=Z_{r}^{}V_{Y}^{H}$, the sparse solution can be then easily recovered from the optimized matrix.

In the case of the standard LASSO algorithm, ${\hat{Z}}_{r}$ is obtained as a solution of the following optimization problem:

(15) $${\hat{Z}}_{r}=\underset{Z_{r}}{\min}C(Z_{r})=\underset{Z_{r}}{\min}\left[\left(1/2\right){\left\|Y_{r}-W^{H}Z_{r}\right\|}_{2}^{2}+\lambda {\left\|Z_{r}\right\|}_{1}\right].$$

The objective function $C(Z_{r})$ is not invariant with respect to data reduction because of the $l_{1}$ norm ${\left\|Z_{r}\right\|}_{1}$. Indeed, ${\left\|Z_{r}\right\|}_{1}={\left\|ZV_{Y}\right\|}_{1}$, which is not equal to ${\left\|Z_{r}\right\|}_{1}$. Hence, it is proposed to replace it with the modified $l_{1}$ norm ${\left\|Z_{r}\right\|}_{1,inv}$, defined as follows:

(16) $${\left\|Z_{r}\right\|}_{1,inv}=Tr\left\{\sqrt{Z_{r}Z_{r}^{H}}\right\}.$$

It can be readily noticed that this newly defined norm is different from the Frobenius norm because of the square root under the trace operator. According to Equations (A4) and (16), it is also data reduction invariant (see Appendix C for further details), so it is called the “invariant $l_{1}$ norm”.

Consequently, if the initial solution (LASSO starting point) belongs to $Fix\left\{Y_{r}\right\}$, the final solution of the data reduction invariant LASSO algorithm can be obtained from:

(17) $${\hat{Z}}_{r}=\underset{Z_{r}}{\min}C_{inv}(Z_{r})=\underset{Z_{r}}{\min}\left[\left(1/2\right){\left\|Y_{r}-W^{H}Z_{r}\right\|}_{2}^{2}+\lambda {\left\|Z_{r}\right\|}_{1,inv}\right].$$

In order to properly describe the LASSO algorithm in its new invariant form, let us consider the following notations:

- $Z_{\left(-i\right)}$: matrix $Z_{r}$ without its *i*th row;
- $Z^{\left(-i\right)}$: the matrix $Z_{r}$ without its *i*th column;
- $Z_{\left(i\right)}$: the *i*th row of the matrix $Z_{r}$;
- $Z^{\left(i\right)}$: the *i*th column of the matrix $Z_{r}$.

One of the basic ideas of the LASSO algorithm is to transform the multidimensional optimization problem (17) into a set of mono-dimensional optimization problems.

This is conducted by expressing the objective function as a sum of two terms, the first one depending on only one component of $Z_{r}$, and the second one depending on all its other components. Thus, the objective function can be optimized successively with respect to each component of $Z_{r}$, which is equivalent to globally optimizing it with respect to all its components.

The problem related to the introduction of the new invariant $l_{1}$ norm ${\left\|Z_{r}\right\|}_{1,inv}$ is that it is not possible anymore to separate a given component of $Z_{r}$ because of the square root function.

By denoting $T=W^{H}$, the following expression holds:

$$R=Y_{r}-W^{H}{\hat{Z}}_{r}=Y_{r}-T{\hat{Z}}_{r}=Y_{r}-\left(T^{\left(-i\right)}{\hat{Z}}_{\left(-i\right)}+T^{\left(i\right)}{\hat{Z}}_{\left(i\right)}\right)$$

(18) $$\Rightarrow R=\left(Y_{r}-T^{\left(-i\right)}{\hat{Z}}_{\left(-i\right)}\right)-T^{\left(i\right)}{\hat{Z}}_{\left(i\right)}.$$

According to the definition of the invariant $l_{1}$ norm, it can be also written as:

(19) $${\left\|{\hat{Z}}_{r}\right\|}_{1,inv}={\left\|{\hat{Z}}_{\left(-i\right)}\right\|}_{1,inv}+{\left\|{\hat{Z}}_{\left(i\right)}\right\|}_{1,inv}={\left\|{\hat{Z}}_{\left(-i\right)}\right\|}_{1,inv}+{\left\|{\hat{Z}}_{\left(i\right)}\right\|}_{2}.$$

Let us also denote:

(20) $$R_{part(i)}=Y_{r}-T^{\left(-i\right)}{\hat{Z}}_{\left(-i\right)}.$$

$C_{inv}(Z_{r})$ then becomes:

Cinv(Zr)=(1/2)‖Rpart(i)−T(i)Z^(i)‖22+λ(‖Z^(−i)‖1,inv+‖Z^(i)‖2)=(1/2)‖Rpart(i)‖22−Tr{Re[Rpart(i)HT(i)Z^(i)]}+(1/2)‖T(i)Z^(i)‖22+λ(‖Z^(−i)‖1,inv+‖Z^(i)‖2)

(21) ⇒Cinv(Zr)=[(1/2)‖Rpart(i)‖22+λ‖Z^(−i)‖1,inv]+[(1/2)‖T(i)Z^(i)‖22+λ‖Z^(i)‖2−Tr{Re[Rpart(i)HT(i)Z^(i)]}].

Focus now only on the second term of $C_{inv}(Z_{r})$ since the first one does not depend on $Z_{\left(i\right)}$. Let us also define the following notations:

(22) $$\begin{array}{l} \mu ={\left\|{\hat{Z}}_{\left(i\right)}\right\|}_{2},\;\tilde{z}={\hat{Z}}_{\left(i\right)}/{\left\|{\hat{Z}}_{\left(i\right)}\right\|}_{2},\\ a=R_{part(i)}^{H}T^{\left(i\right)},\;b=T^{\left(i\right)}. \end{array}$$

Hence, the objective function becomes:

(23) Cinv(z˜,μ)=(1/2)μ2‖bz˜‖22+λμ−μRe[z˜a].

The value of *µ* minimizing $C_{inv}(Z_{r})$ can be then obtained from:

(24) ∂Cinv(z˜)∂μ=0⇒μ=Re[z˜a]−λ‖bz˜‖22.

If Equation (24) yields a negative value for *µ*, take μ=0 since μ≥0 according to Equation (22).

For a fixed value of *µ*, by keeping only the terms depending on $\tilde{z}$, the objective function to be minimized with respect to $\tilde{z}$ can be written as:

(25) C′inv(z˜,μ)=(1/2)μ‖bz˜‖22−Re[z˜a],subject to: ‖z˜‖22−1=0.

Lagrange’s multipliers method leads to the following objective function:

(26) F(z˜,θ)=(1/2)μ‖bz˜‖22−Re[z˜a]+(θ/2)(‖z˜‖22−1).

where *θ* stands for the Lagrange multiplier.

Developing Equation (26) to make the components of $\tilde{z}$ appear results in:

(27) F(z˜1,z˜2,…,z˜M,θ)=(1/2)μ∑k∑l|bk|2|z˜l|2− ∑lRe[alz˜l]+(θ/2)(∑l|z˜l|2−1)=(1/2)μ‖b‖22∑l|z˜l|2− ∑lRe[alz˜l]+(θ/2)(∑l|z˜l|2−1).

The phase ${\tilde{z}}_{l}$ is involved only in the product $a_{l}{\tilde{z}}_{l}$, and it can be readily seen that *F* is minimized when:

(28) $$\arg\left\{{\tilde{z}}_{l}\right\}=-\arg\left\{a_{l}\right\},$$

so that:

(29) $$F({\tilde{z}}_{1},{\tilde{z}}_{2},\ldots ,{\tilde{z}}_{M},\theta )=\left(1/2\right)\mu {\left\|b\right\|}_{2}^{2}\sum_{l}{\left|{\tilde{z}}_{l}\right|}^{2}-\;\sum_{l}\left|a_{l}\right|\left|{\tilde{z}}_{l}\right|+\left(\theta /2\right)\left(\sum_{l}{\left|{\tilde{z}}_{l}\right|}^{2}-1\right).$$

The value of $\left|{\tilde{z}}_{l}\right|$ that minimizes *F* is then obtained from:

$$\frac{\partial F}{\partial \left|{\tilde{z}}_{l}\right|}=0\Rightarrow \mu {\left\|b\right\|}_{2}^{2}\left|{\tilde{z}}_{l}\right|-\left|a_{l}\right|+\theta \left|{\tilde{z}}_{l}\right|=0$$

(30) $$\Rightarrow \left|{\tilde{z}}_{l}\right|=\frac{\left|a_{l}\right|}{\mu {\left\|b\right\|}_{2}^{2}+\theta }.$$

From Equations (28) and (30), it can be inferred that:

(31) $$\tilde{z}=\eta a^{H},$$

and because $\tilde{z}$ is a unit vector, it can be finally expressed as:

(32) $$\tilde{z}=a^{H}/{\left\|a\right\|}_{2}.$$

In practice, $\tilde{z}$ is first calculated using Equation (32), then *µ* is evaluated from Equation (24); the value of *λ* is estimated using the cross-validation method [30]. Finally, ${\hat{Z}}_{\left(i\right)}=\mu \tilde{z}$ is computed from Equation (22).

Algorithm 1 below summarizes the processing flow associated with the proposed data reduction invariant LASSO technique.

**Algorithm 1** Processing flow for the proposed data reduction invariant LASSO technique
**Input**: *M* × *N* matrix Y at the output of the MWC scheme, *M* × *L* X sampling-related matrix $T=W^{H}$ and *N* × *M* matrix $V_{Y}$, obtained from the SVD of the Y matrix using Equation (3).
**Initialization**:
  Compute $Y_{r}=YV_{Y}$ according to Equation (2).
  Take an initial solution for the *L* × *M* matrix ${\hat{Z}}_{r}$ belonging to $Fix\left\{Y_{r}\right\}$.
  Find the optimal λ value using the cross-validation method [30].
**For***i*←1 to *L*, **do**
  Obtain the matrices $T^{\left(-i\right)}$ and ${\hat{Z}}_{\left(-i\right)}$ by removing the *i*th column from the matrix T and the *i*th row from the matrix ${\hat{Z}}_{r}$, respectively. 
  In addition, obtain the *i*th column of matrix T and denote it by $b=T^{\left(i\right)}$.
  Calculate $R_{part(i)}=Y_{r}-T^{\left(-i\right)}{\hat{Z}}_{\left(-i\right)}$ according to Equation (20).
  Calculate $a=R_{part(i)}^{H}T^{\left(i\right)}$ according to Equation (22).
  Calculate $\tilde{z}=a^{H}/{\left\|a\right\|}_{2}^{}$ according to Equation (32) and then μ=Re[z˜a]−λ‖bz˜‖22 according to Equation (24).
  Calculate ${\hat{Z}}_{\left(i\right)}=\mu \tilde{z}$ according to Equation (22).
  Update the estimated solution ${\hat{Z}}_{r}$ by replacing its *i*th row with ${\hat{Z}}_{\left(i\right)}$.

**End for**

**Output**: Calculate the final estimated solution, i.e., the *L* × *N* matrix $\hat{Z}={\hat{Z}}_{r}V_{Y}^{H}$.


A comparison of complexity can be finally performed between the proposed algorithm and the standard one. Thus, based on Algorithm 1 presented above, it can be readily established that the complexity is reduced from $O\left(MNL^{2}\right)$ for the standard LASSO to O(MN(L+M)) for the data invariant LASSO algorithm. It can be noticed that the complexity gain increases with the value of *L* since the proposed algorithm reduces its quadratic dependence on this parameter to a linear one. An additional complexity result is provided in the next section in terms of the number of multiplications for a given set of simulation parameters.

## 4. Simulation Results

This section aims to illustrate the performance of the proposed invariant LASSO algorithm in a simulated wideband spectrum sensing scenario characterized by the following parameters:

- Monitored frequency band: −1 GHz≤ν≤1 GHz;
- The number of active frequency bands: $N_{b}=8$;
- Bandwidth of each active frequency band: B=20 MHz;
- Spectral resolution: Δν=30.518 kHz;
- The number of MWC parallel processing paths M=21;
- Sampling frequency on each path: $F_{s}=F_{p}=31.25\;\mathrm{MHz}$;
- The number of samples acquired on each path N=1024.

Note that for this set of parameters, the sizes of the matrices Y and Z are lowered from 21 × 1024 and 32 × 1024, to 21 × 21 and 32 × 21, respectively. It can be also noticed that given the real nature of the analyzed signal, the eight active frequency bands have to be considered by couples of two so that they actually correspond to four transmitters.

**Figure 2:**
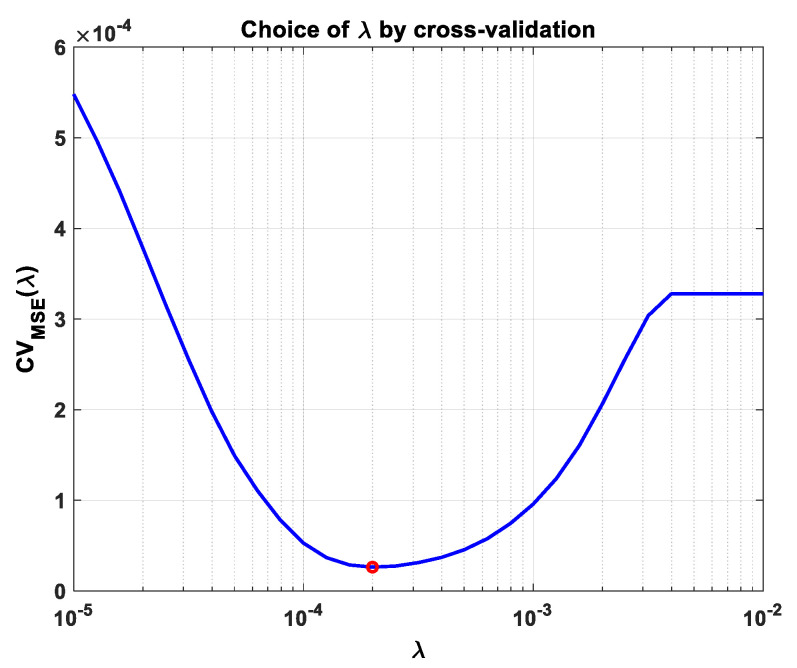
Choice of optimal *λ* value (red circle) using the cross-validation method.

Figure 2 shows the variation in the cost function during the cross-validation process. Its minimum value is obtained for $\lambda ≅2\cdot 10^{-4}$. This value of *λ* does not depend on the noise level and is used by the invariant LASSO algorithm, as explained in the previous section.

**Figure 3:**
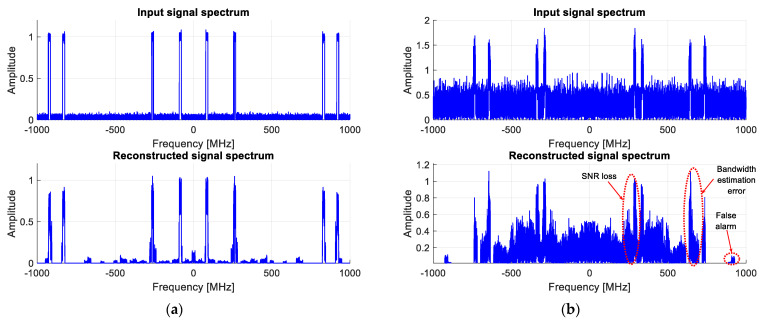
Wideband spectrum sensing results obtained using the invariant LASSO algorithm, for an In-band SNR of (**a**) 30 dB; (**b**) 10 dB.

Figure 3 illustrates the new algorithm performance for two signal-to-noise ratios (SNR), i.e., 30 dB and 10 dB, respectively. Note that these two values are in-band SNRs since they are measured only within the active bands. If the same SNRs are calculated over the whole monitored band, they correspond to 19 dB and −1 dB, respectively.

As can be readily seen, the active bands are very well reconstructed for an in-band SNR = 30 dB, and they can be perfectly detected using an appropriate threshold, which is iteratively and blindly updated, as has also been already proposed in [31].

For an in-band SNR = 10 dB, although the results are still exploitable, the algorithm reaches its limits. This can be explained by the fact that the LASSO algorithm introduces an SNR loss in the reconstructed bands of about 11 dB in this configuration. Indeed, as can be noticed from Figure 3b, SNR loss leads to the increasingly challenging detection of active bands, as well as higher false alarm rates and bandwidth estimation errors.

**Figure 4:**
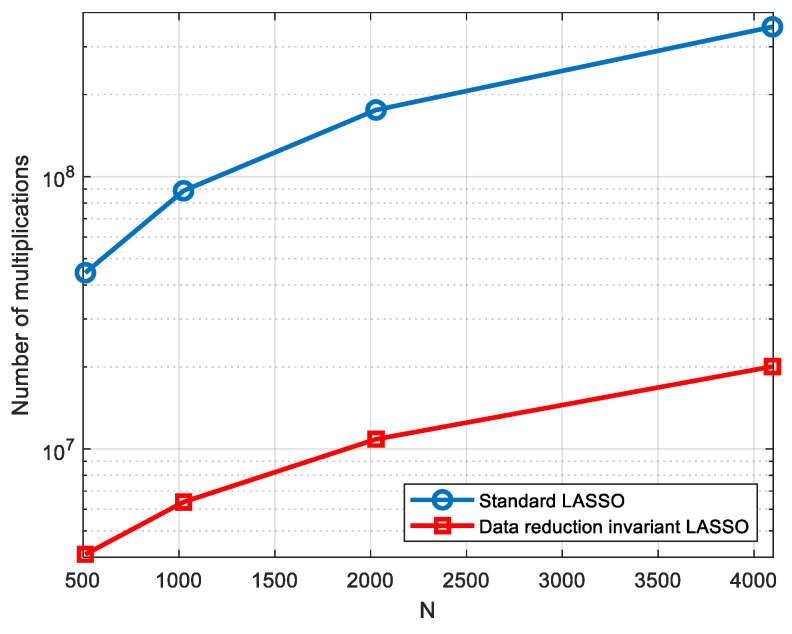
Complexity gain comparison in terms of number of multiplications.

In order to evaluate the complexity gain for the considered set of simulation parameters (*M* = 21, *L* = 32), the number of multiplications is also shown in Figure 4 for N∈{512,1024,2048,4096}. It can be noticed that a significant complexity reduction is obtained using the proposed algorithm, which becomes even slightly larger with the value of *N*.

**Figure 5:**
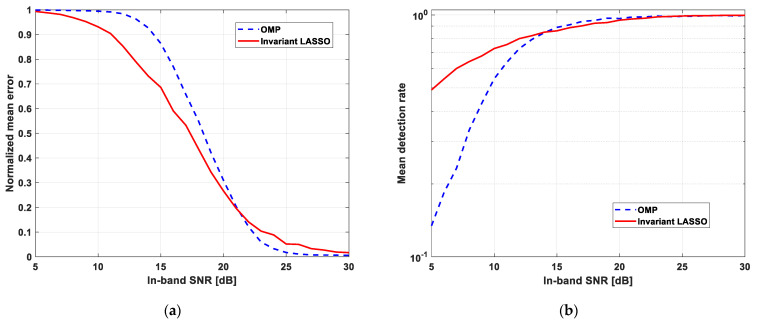
Performance comparison between OMP and LASSO algorithms in terms of: (**a**) Normalized mean error; (**b**) Mean detection rate.

The performance of the new algorithm was finally evaluated for a wide range of in-band SNR (5–30 dB) and false alarm probabilities ($10^{-6}-10^{-1}$) in terms of the normalized mean error and detection rate (Figure 5). The same parameters provided by the OMP algorithm were also plotted for comparison purposes.

These two performance parameters have been obtained at the output of a “threshold and detect” scheme, using Monte-Carlo simulations with 1000 independent noise realizations and random positions of the active frequency bands. The threshold is calculated to keep the false alarm rate constant at the output of this scheme. The normalized error is then obtained as a complement with respect to one of the relative numbers of threshold overruns inside the active frequency bands. The detection rate is calculated as the relative number of detected frequency bands. Note that a frequency band is considered as being detected if there is at least one threshold overrun inside it.

The results depicted here have been obtained for a false alarm probability of $10^{-3}$, but they are similar to the other false alarm probabilities in the range above. It can be noticed that the performances of the two algorithms are close. However, the proposed algorithm appears to be more robust to noise, while OMP provides a slightly better detection rate for high SNRs.

## 5. Experimental Results

**Figure 6:**
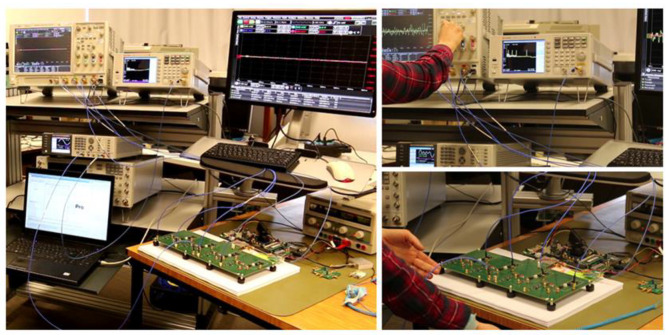
Experimental testbed for the evaluation of data reduction invariant LASSO algorithm.

**Figure 7:**
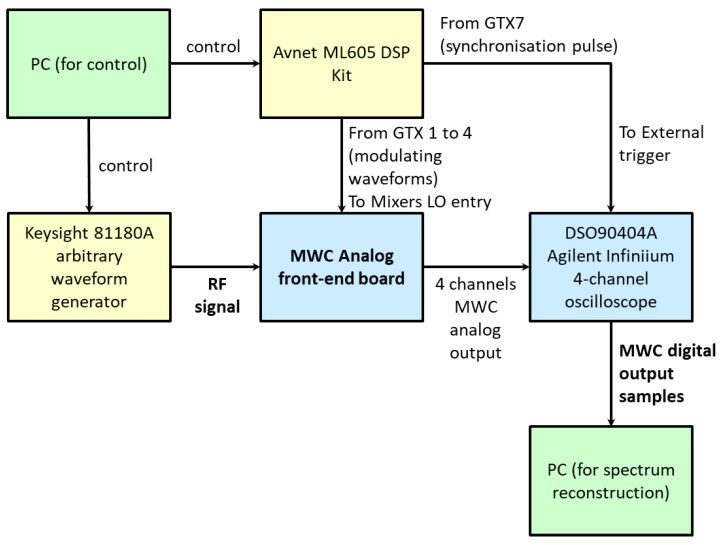
Block diagram of our compressed sampling testbed.

The proposed data reduction invariant LASSO was also evaluated using measured data. Our experimental testbed is shown in Figure 6, and its block diagram, including the external instruments, is provided in Figure 7. It is based on a 4-channels MWC analog board, which is described in more detail in a previously published paper [13], and is able to monitor wideband spectral domains up to 1 GHz. Table 1 provides the main parameters of our experimental testbed.

**Figure 8:**
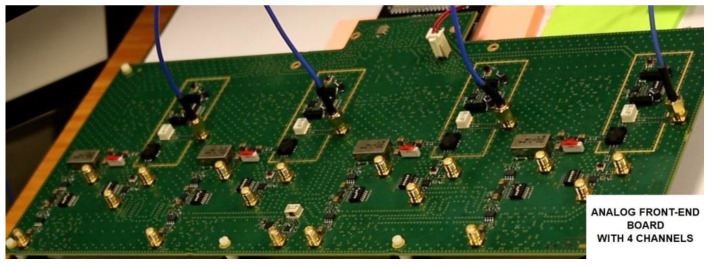
Photo of our analog front-end board.

**Figure 9:**
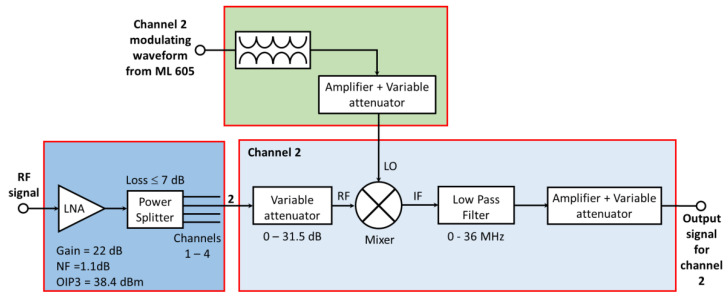
Illustration of the operating principle of our analog front-end board.

Our analog front-end board for compressed sampling with its four physical channels is shown in Figure 8, while Figure 9 illustrates its operating principle. On input, an SCA-4-10+ splitter from Mini-Circuits©, with less than 7 dB loss, was used to provide the input signal to the four channels.

Similar to channel 2 depicted in Figure 9, each channel included an M1-0008 mixer from MArki©. The mixer receives an amplified radio-frequency signal to analyze its RF input and a pseudo-random modulating waveform on its LO input.

The mixer output (IF) goes through a low-pass filter, which is an SXLP-36+ low-pass filter from Mini-Circuits©, with a 3 dB cut-off frequency of 40 MHz. This filter was chosen because it has a very flat response (variations lower than 1 dB) from the 0 to 36 MHz band and a sharp cut-off above this band.

**Figure 10:**
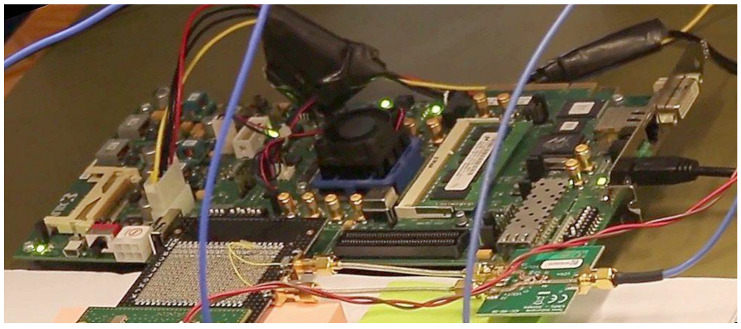
Avnet ML605 DSP Kit used for the generation of modulating waveforms.

For our experiments, the radio-frequency signal was provided by a Keysight 81180A arbitrary waveform generator. An Avnet ML605 DSP Kit, as shown in Figure 10, was also used to generate the pseudo-random modulating waveforms. It included a Xilinx Virtex-6 FPGA, as well as digital-to-analog and analog-to-digital capabilities. Moreover, it enables the selection of each channel sequence from a compiled list. If necessary, recompilation allows new sequences to be added or changes some parameters, such as the bit rate. The embedded Gigabit-Transceiver X (GTX) high-speed Serializer-Deserializer transceivers from 0 to 3 were connected to channels 1–4 of the front-end analog board.

A DSO90404A Agilent Infiniium 4-channel oscilloscope was used to acquire the output signals and save them. To synchronize the acquisition with respect to the modulating waveforms, a pulse signal was generated by the GTX 7 of the ML605 board and plugged into the oscilloscope external trigger.

**Figure 11:**
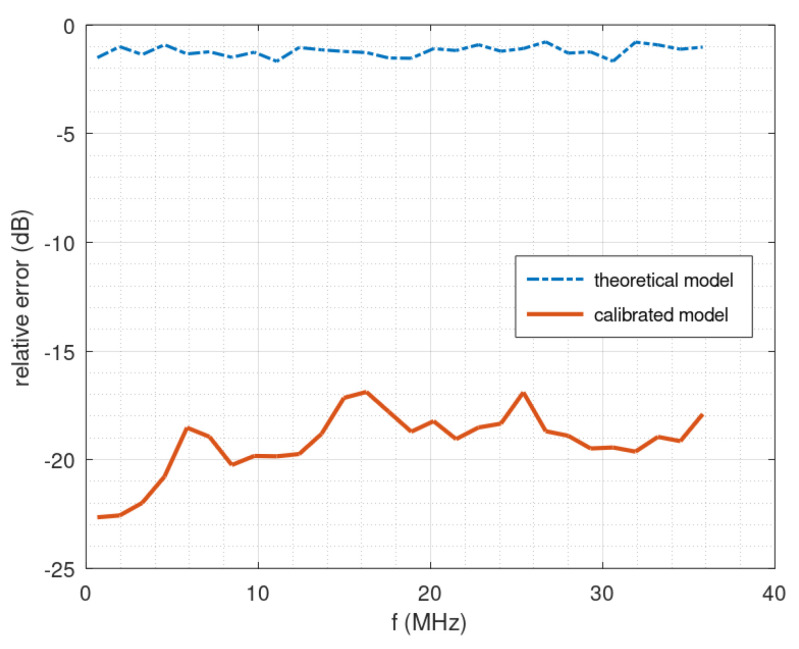
Relative error between observed and predicted system output, as a function of output frequency.

The acquisition system was calibrated using the approach described in [32]. Figure 11 shows the relative error between the observed and predicted system output, as a function of output frequency, for the calibrated system and for the uncalibrated one, which clearly demonstrates the interest in the calibration stage. The relative error was evaluated using a formula similar to the criterion considered in [33]:

(33) $$\varepsilon (f)=20\log_{10}\left(\frac{\left\|o(f)-p(f)\right\|}{\left\|o(f)\right\|}\right).$$

Here *o*(*f*) denotes the observed output signal corresponding to the subband centered on *f* (the whole frequency band of the signal at the output of the acquisition system has been divided into 28 subbands). Similarly, *p*(*f*) denotes the predicted output signal corresponding to the subband centered on *f*, which is predicted by the calibrated model or by the theoretical model. In any case, even for the theoretical model, a calibrated low-pass filter is always included: the true frequency response of the filter is taken into account in the related equations.

**Figure 12:**
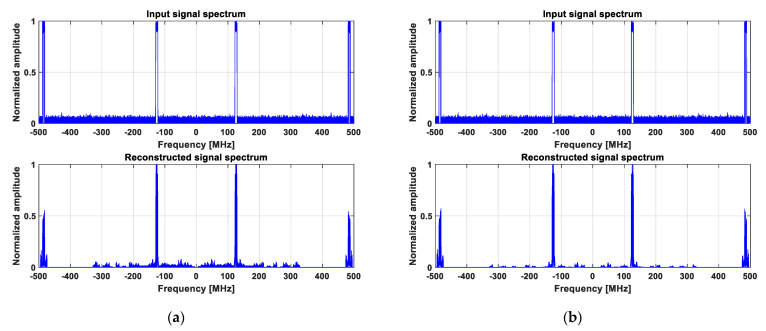
Wideband spectrum sensing results provided by the proposed reconstruction algorithm for two active transmitters: (**a**) Original measured data; (**b**) Reduced data.

**Figure 13:**
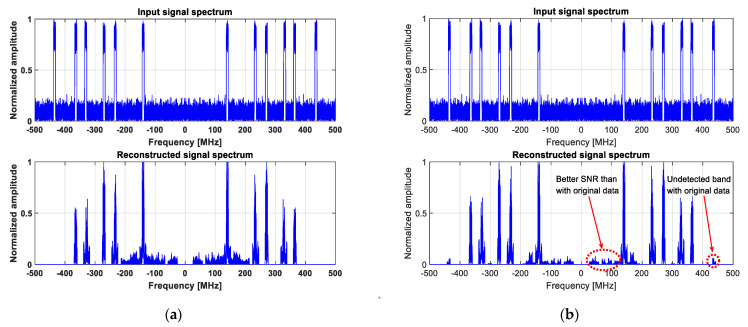
Wideband spectrum sensing results provided by the proposed reconstruction algorithm for six active transmitters: (**a**) Original measured data; (**b**) Reduced data.

The wideband spectrum sensing results provided by the proposed reconstruction algorithm, using original and reduced data, are shown in Figure 12 and Figure 13 for 2 and 6 active transmitters, respectively.

For the scenario when two active transmitters, i.e., four active frequency bands, are considered (Figure 12), it can be noticed that they are both well detected. The amplitude of the upper-frequency bands is lower than expected because the corresponding transmitter carrier is close to the higher limit of the monitored frequency band. We have noticed that the reconstruction is usually less reliable in this area, probably due to higher non-linear effects in the analog front-end at very high frequencies, and it is interesting to see that the transmitter is detected even in these difficult conditions.

For the scenario with six active transmitters, i.e., 12 active frequency bands (Figure 13), the first five transmitters are well detected, while the last one seems to be lost. In addition to the fact that it is also close to the higher limit of the monitored frequency band, as in the previous case, there is another aspect that explains this result. Actually, with six transmitters to be detected instead of two, the expected solution is significantly less sparse than in the previous case, which leads to some reconstruction quality loss.

However, it can be readily seen that the reconstruction results are slightly better, in terms of SNR and MSE, when the proposed algorithm runs on reduced data, as shown in Figure 13b. The execution time is also about 20 times shorter than when it runs on original data, which confirms the results presented in Figure 4.

**Figure 14:**
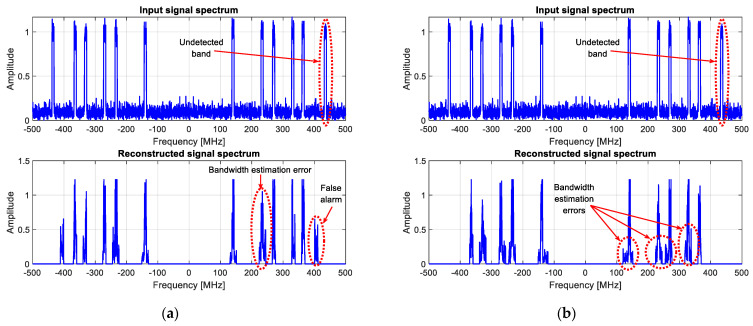
Wideband spectrum sensing results for six active transmitters obtained using two greedy algorithms: (**a**) CoSaMP; (**b**) IHT.

Finally, as already mentioned in Section 2, the reconstruction results obtained with two greedy algorithms, CoSaMP and IHT, are also illustrated in Figure 14 for the same measured data scenario with six transmitters. Note that there seems to be less noise on these images just because, contrariwise to LASSO, in the case of greedy algorithms, the spectrum is reconstructed only inside the detected active bands. However, as illustrated in Figure 14, they are more likely to miss some transmitters and generate false alarms. They can also be subject to bandwidth estimation errors if further processing is carried out to extract more information related to the detected bands. Note that this kind of post-processing is out of the scope of the paper and is mentioned here only to illustrate the limitations of CoSaMP and IHT algorithms.

## 6. Conclusions

This paper introduces a new idea for designing a highly effective wideband spectrum sensing system, which consists of reducing the data matrix at the output of the Xsampling MWC scheme. The second contribution of this paper is the demonstration of data reduction invariance on greedy sparse reconstruction algorithms. Finally, our most important contribution presented in this paper is a new version of the LASSO algorithm, which is also invariant with respect to the same criterion. Coupled with the data reduction idea, the proposed algorithm is a powerful and effective tool in the wideband spectrum sensing framework, especially for low SNR values.

As a future work, the newly proposed method should be tested and further improved in the presence of impulsive noise, as has been already conducted in [34] for greedy algorithms.

## Figures and Tables

**Table 1:** Main parameters of the experimental testbed used to evaluate the proposed algorithm.

Parameter	Symbol	Value
Number of physical channels	M	4
Nyquist frequency	$\;F_{Nyq}$	1 GHz
Length of scramblers	L	96
Scramblers repetition frequency	$\;F_{p}=F_{Nyq}/L$	10.41667 MHz
ADC sampling frequency	$\;F_{s}$	104.1667 MHz
Measured samples/channel	N	448
Spectral resolution	$\Delta \nu =F_{Nyq}/\left(L\times N\right)$	23.251 kHz

## Appendix A

**Lemma** **A1.**
*The following equality holds:*

(A1)
Y∈Fix{Y}.

**Proof.** Indeed, $Y\Pi_{Y}=U_{Y}S_{Y}\underset{I_{M}}{\underset{︸}{V_{Y}^{H}V_{Y}}}V_{Y}^{H}=Y$. □

**Lemma** **A2.** A∈Fix{Y} ⇔ *there exists a matrix* $A_{r}$ *(not a necessary square) such that:*

(A2) $$A=A_{r}V_{Y}^{H}.$$

*In the following,* $A_{r}$ *is said to be a reduced form of* **A***, because in our application* $A_{r}$ *is considerably smaller than* **A**.

**Proof.** If $A=A_{r}V_{Y}^{H}$, then $A\Pi_{Y}=A_{r}\underset{I_{M}}{\underset{︸}{V_{Y}^{H}V_{Y}}}V_{Y}^{H}=A$. In the other sense, if A∈Fix{Y}, then $AV_{Y}V_{Y}^{H}=A\Rightarrow A_{r}=AV_{Y}$. □

**Lemma** **A3.** *If*A,B∈Fix{Y}, *then* ∀ $\Lambda_{A}$ *and* $\Lambda_{B}$ *(not necessary square, nor diagonal), and the following equality holds:*

(A3) $$\Lambda_{A}A+\Lambda_{B}B\in Fix\left\{Y\right\}.$$

**Proof.** $\Lambda_{A}A+\Lambda_{B}B=\Lambda_{A}A_{r}V_{Y}^{H}+\Lambda_{B}A_{r}V_{Y}^{H}\Rightarrow \Lambda_{A}A+\Lambda_{B}B=\left(\Lambda_{A}A_{r}+\Lambda_{B}A_{r}\right)V_{Y}^{H}$, so Equation (A3) is true according to Lemma A2. □

**Lemma** **A4.**
*If* A∈Fix{Y} *and* $A_{r}$ *is a reduced form of* **A** *, then for any norm, which can be expressed by:*

(A4)
$$\left\|A\right\|=f\left(AA^{H}\right),$$

*with f any continuous function, the following equality holds:*

(A5)
$$\left\|A\right\|=\left\|A_{r}\right\|.$$

**Proof.** Indeed, $\left\|A\right\|=f\left(AA^{H}\right)=f\left(A_{r}\underset{I_{M}}{\underset{︸}{V_{Y}^{H}V_{Y}}}A_{r}^{H}\right)=f\left(A_{r}A_{r}^{H}\right)=\left\|A_{r}\right\|$. □

**Corollary** **A1.**
*The Frobenius norm meets Equation (A5), i.e.:*

(A6)
$${\left\|A\right\|}_{F}={\left\|A_{r}\right\|}_{F}.$$

**Proof.** The proof of this corollary is straightforward since the Frobenius norm ${\left\|A\right\|}_{F}=\sqrt{Tr\left\{AA^{H}\right\}}$ already has the form required in Equation (A4). □

## Appendix B

OMP is a greedy algorithm that is widely used for finding the sparse solution of the problem described by Equation (1). For the sake of simplicity and without any loss of generality, its operating principle is recalled hereafter for the case when the measured input data **y** and the expected sparse solution **z** are vectors instead of matrices.

Let us introduce the following notations related to the *k*th iteration: ${\hat{z}}_{k}$ for the estimated vector solution, $r_{k}=y-W^{H}{\hat{z}}_{k}$ for the residual vector, $i_{k}$ for the index vector of ${\hat{z}}_{k}$ non-zero components, ${\underline{\hat{z}}}_{k}={\hat{z}}_{k}(i_{k})$ for the estimated vector reduced to its non-zero components, and ${\underline{W}}_{k}^{H}=W^{H}(:,i_{k})$ for the matrix formed with the columns of $W^{H}$ corresponding to the indices contained in $i_{k}$.

Basically, OMP starts with a null solution and adds a new non-zero component at each iteration so that, to minimize the norm of the residual vector $r_{k}$, which measures the reconstruction error, the algorithm stops when the targeted number of non-zero components is reached or when the residual norm is below a given threshold.

Since $W_{}^{H}{\hat{z}}_{k}={\underline{W}}_{k}^{H}{\underline{\hat{z}}}_{k}$, the residual vector can be also written as $r_{k}=y-{\underline{W}}_{k}^{H}{\underline{\hat{z}}}_{k}$. It is then possible to place the problem at the *k*th iteration in the following equivalent form:

(A7) $$\underset{{\hat{z}}_{k}}{\min}{\left\|y-W_{}^{H}{\hat{z}}_{k}\right\|}_{}^{2}=\underset{{\underline{\hat{z}}}_{k}}{\min}{\left\|y-{\underline{W}}_{k}^{H}{\underline{\hat{z}}}_{k}\right\|}_{}^{2}.$$

The advantage of this equivalent form is that, contrary to the initial problem, it results in the unique solution:

(A8) $${\underline{\hat{z}}}_{k}={\underline{W}}_{k}^{†}y,$$

where ${\underline{W}}_{k}^{†}$ is the Moore–Penrose pseudo-inverse of ${\underline{W}}_{k}^{H}$.

Note that ${\hat{z}}_{k}$ can be obtained from ${\underline{\hat{z}}}_{k}$, provided that $i_{k}$ is known. Actually, only the last component of the vector $i_{k}$ has to be determined at the *k*th iteration, the other k−1 components being found out during the previous iterations.

Keeping in mind that the OMP algorithm aims at minimizing the residual norm ${\left\|r_{k}\right\|}^{2}$, and since the product $W_{}^{H}{\hat{z}}_{k}$ can be seen as the linear combination of the columns of $W^{H}$ matrix weighted by the non-zero components of ${\hat{z}}_{k}$, the *k*th component of $i_{k}$ can be determined as follows:

(A9) $$i_{kk}=\arg\underset{l}{\max}\left(\frac{\left|\langle r_{k-1},w_{l}\rangle \right|}{\left\|r_{k-1}\right\|\cdot \left\|w_{l}\right\|}\right),$$

where $\langle r_{k-1},w_{l}\rangle$ stands for the scalar product between the residual vector at the ${\left(k-1\right)}^{th}$ iteration and the *l*th column of the matrix $W^{H}$.

In other words, the newly added non-zero component of the vector ${\hat{z}}_{k}$ corresponds to the most correlated column of the matrix $W^{H}$ with the residual vector $r_{k-1}$. Note that all the non-zero components of the vector ${\hat{z}}_{k}$ are updated using Equation (A8).

The convergence of the OMP algorithm toward the global optimum is ensured in the noiseless case, for a random $W^{H}$ matrix and provided that M≥2slog(L), where *M* is the length of the measured vector **y**, *L* is the length of the sparse vector **z**, and *s* is the number of its non-zero components.

## Appendix C

This appendix explains in more detail why the modified $l_{1}$ norm ${\left\|\cdot \right\|}_{1,inv}$ is invariant with respect to data reduction.

Let us first consider the case of a 2 × 2 matrix **Z**. According to Equation (15), its modified $l_{1}$ norm can be calculated as follows:

(A10) $$\begin{array}{l} {\left\|Z\right\|}_{1,inv}=Tr\left\{\sqrt{ZZ^{H}}\right\}=Tr\left\{\sqrt{\left[\begin{array}{cc} z_{11} & z_{12}\\ z_{21} & z_{22} \end{array}\right]\left[\begin{array}{cc} z_{11}^{*} & z_{21}^{*}\\ z_{12}^{*} & z_{22}^{*} \end{array}\right]}\right\}\\ =Tr\left\{\sqrt{\left[\begin{array}{cc} {\left|z_{11}\right|}^{2}+{\left|z_{12}\right|}^{2} & z_{11}z_{21}^{*}+z_{12}z_{22}^{*}\\ z_{21}z_{11}^{*}+z_{22}z_{12}^{*} & {\left|z_{21}\right|}^{2}+{\left|z_{22}\right|}^{2} \end{array}\right]}\right\}=\sqrt{{\left|z_{11}\right|}^{2}+{\left|z_{12}\right|}^{2}}+\sqrt{{\left|z_{21}\right|}^{2}+{\left|z_{22}\right|}^{2}}. \end{array}$$

It can be readily seen that if the matrix **Z** becomes a vector $Z={\left[\begin{array}{cc} z_{11} & z_{21} \end{array}\right]}^{T}$, then its modified $l_{1}$ norm results in the standard $l_{1}$ norm:

(A11) $${\left\|Z\right\|}_{1,inv}=\sqrt{{\left|z_{11}\right|}^{2}}+\sqrt{{\left|z_{21}\right|}^{2}}=\left|z_{11}\right|+\left|z_{21}\right|={\left\|Z\right\|}_{1}.$$

Consequently, the new defined invariant $l_{1}$ norm is equivalent to the standard $l_{1}$ norm whenever **Z** is a vector, so that it is able to take into account the sparsity of the searched solution.

In the case of a *M* × *N* matrix **Z**, the invariant $l_{1}$ norm takes the form:

$${\left\|Z\right\|}_{1,inv}=\sqrt{{\left|z_{11}\right|}^{2}+\cdots +{\left|z_{1N}\right|}^{2}}+\cdots +\sqrt{{\left|z_{M1}\right|}^{2}+\cdots +{\left|z_{MN}\right|}^{2}}$$

(A12) $$\Rightarrow {\left\|Z\right\|}_{1,inv}={\left\|Z_{\left(1\right)}\right\|}_{2}+\cdots +{\left\|Z_{\left(M\right)}\right\|}_{2}.$$

where $Z_{\left(i\right)}$ denotes the *i*th row of matrix **Z**.

Just for comparison, the standard $l_{1}$ norm, in this case, yields:

$${\left\|Z\right\|}_{1}=\left|z_{11}\right|+\left|z_{12}\right|\cdots +\left|z_{1N}\right|+\cdots +\left|z_{M1}\right|+\left|z_{M2}\right|\cdots +\left|z_{MN}\right|$$

(A13) $$\Rightarrow {\left\|Z\right\|}_{1}={\left\|Z_{\left(1\right)}\right\|}_{1}+\cdots +{\left\|Z_{\left(M\right)}\right\|}_{1}.$$

Once again, it can be readily seen that when **Z** is a vector (i.e., each of its rows become a scalar) ${\left\|Z\right\|}_{1,inv}={\left\|Z\right\|}_{1}$, otherwise ${\left\|Z\right\|}_{1,inv}\neq {\left\|Z\right\|}_{1}$.

However, while ${\left\|Z\right\|}_{1}$ is not invariant with respect to data reduction, ${\left\|Z\right\|}_{1,inv}$ has this property according to Equations (A4) and (16).
